# Self-Control Capacity Moderates the Effect of Stereotype Threat on Female University Students’ Worry During a Math Performance Situation

**DOI:** 10.3389/fpsyg.2022.794896

**Published:** 2022-04-07

**Authors:** Alex Bertrams, Christoph Lindner, Francesca Muntoni, Jan Retelsdorf

**Affiliations:** ^1^Educational Psychology Lab, Institute of Educational Science, University of Bern, Bern, Switzerland; ^2^Faculty of Education, University of Hamburg, Hamburg, Germany; ^3^Leibniz Institute for Science and Mathematics Education, Kiel, Germany

**Keywords:** gender, self-control capacity, self-regulation, stereotype threat, test anxiety, worry

## Abstract

Stereotype threat is a possible reason for difficulties faced by girls and women in the fields of science, technology, engineering, and mathematics. The threat experienced due to gender can cause elevated worry during performance situations. That is, if the stereotype that women are not as good as men in math becomes salient, this stereotype activation draws women’s attention to task-irrelevant worry caused by the fear of conforming to the negative stereotype. Increased worry can reduce cognitive resources, potentially leading to performance decrements. We argue that such worry is more pronounced immediately after an unrelated self-control demand, which is assumed to temporarily decrease people’s self-control exertion over their attention and stream of thought (i.e., relatively low self-control capacity). This prediction was examined in an experiment conducted with 102 participating university students enrolled in courses in which math plays a crucial role. After the manipulation of self-control capacity (low vs. high), stereotype threat was induced for the female students, but not the male students. Then, the students were asked to report their thoughts during a math performance situation (i.e., written thought protocols) three times. Multiple-group autoregressive path models revealed that when self-control capacity was relatively low, female compared with male students reported more intense worry in the initial two thought protocols. In contrast, in the relatively high self-control capacity condition, female and male students did not differ significantly in their reported worry at any time. These results expand on previous findings, suggesting that threat effects depend on definable situational self-control conditions.

## Introduction

The underrepresentation of girls and women in the science, technology, engineering, and mathematics (STEM) fields continues to be a concern not only for educators and scientists, but also for society. Gender differences in mathematics and science achievement have been reported in the literature over many decades (Hedges and Nowell, 1995; Reilly et al., 2019). Furthermore, boys also report more positive attitudes toward learning mathematics and science (Else-Quest et al., 2010), and girls report lower self-confidence and greater mathematics anxiety (Beilock et al., 2010). However, researchers are divided on how significant gender differences in mathematics and science are. Some argue that the differences are small but still meaningful (Reilly et al., 2015), while others argue that these differences are very minimal on average or do not exist (Hyde, 2005; Else-Quest et al., 2010). For example, the gender similarities hypothesis, proposed by Hyde (2005), states that males and females are similar on most, but not all, psychological variables, and meta-analytic evidence suggests that girls and boys do not, in fact, perform differently in measures of mathematics achievement (Lindberg et al., 2010). Nevertheless, from an early age, children show rigid gender stereotyping, perceiving mathematics and science as male domains (Plante et al., 2009; Martinot et al., 2012). These negative stereotypes can affect girls’ mathematics performance through the mechanism of stereotype threat (Evans et al., 2011; Tomasetto et al., 2011; Passolunghi et al., 2014). Moreover, it has been found that these gender stereotypes persist into adulthood and are a cross-cultural phenomenon (Nosek et al., 2002).

Stereotype threat has frequently been studied as a possible reason for the weaker engagement, motivation, and performance of girls and women in STEM (Pennington et al., 2016; Spencer et al., 2016). The stereotype that females are less able than males in STEM tasks can cause fear of confirming the stereotype, leading to task-irrelevant worry and even performance degradation in STEM test situations (Schmader and Johns, 2003). To the best of our knowledge, no study has yet examined the moderating situational circumstances of the stereotype threat effect on worry. Addressing this lacuna, in this study, we examine whether worry is more pronounced immediately after an unrelated effortful self-control demand which causes relatively low momentary self-control capacity. To do so, we drew on a university student sample enrolled in courses in which math plays a crucial role. The current study allowed us to extend previous research in at least two ways. First, in addition to previous research focusing on the relation between stereotype threat and worry (e.g., Cadinu et al., 2005), we considered the possible moderating function of students’ self-control capacity. Second, this study augments the existing research on test anxiety and self-control capacity (e.g., Bertrams et al., 2013) by investigating the effect of another origin of threat (i.e., being stereotyped).

### Stereotype Threat and the Role of Worry

A growing body of literature has investigated the relation between stereotypes and performance. The most significant work in this area is the research on *stereotype threat*, which indicates that negative stereotypes hamper the academic performance of stereotyped individuals (Steele and Aronson, 1995; Tempel and Neumann, 2014; Pennington et al., 2016; Spencer et al., 2016). The stereotype threat effect has been characterized as a “psychological predicament in which individuals are inhibited from performing to their potential by the recognition that possible failure could confirm a negative stereotype that applies to their ingroup and, by extension, to themselves” (Schmader, 2002, p. 194). Thus, stereotype threat is viewed as a form of social identity threat.

In their influential paper, Steele and Aronson (1995) noted that the stereotype that African American students have lower academic ability hampered the performance of African American students on academic tests. As African Americans are well aware of the negative stereotypes questioning their intellectual ability, they might fear confirming this stereotype. This fear of stereotype confirmation is considered to occupy the cognitive systems required for optimal performance and therefore leads to low test performance. This study has stimulated numerous subsequent studies investigating the influence of negative stereotypes. For example, when confronted with the negative stereotype about their in-group, women were found to underperform on math tests (e.g., Spencer et al., 1999) and driving tests (Yeung and von Hippel, 2008), older adults were found to underperform on memory tests and cognitive tests (Lamont et al., 2015), and students with lower socio-economic status were found to underperform on intelligence tests (Désert et al., 2009). However, of the many negative stereotypes that have been studied in the context of stereotype threat, the stereotype that women are not as good in mathematics as men is one of the most frequently studied (e.g., Spencer et al., 1999; Schmader, 2002; Tomasetto et al., 2011; Tomasetto, 2019). Research on this topic has shown that women and girls exhibit lower math performance if they are reminded of the negative stereotype about girls and their inferior math ability, but they perform as well as boys if such stereotypes are not made salient before they take a math test.

However, what causes performance declines in stereotype-threatening situations? Stereotype threats do not generally lead to decreased motivation in performance situations. Instead, people experiencing stereotype threat are motivated to disprove negative stereotypes about their social identity (e.g., Nussbaum and Steele, 2007; Vandello et al., 2008) or at least to avoid confirming them (e.g., Brodish and Devine, 2009; Chalabaev et al., 2012). Thus, stereotype threat creates the desire to do well on a given task and disprove the negative stereotypes (Steele and Aronson, 1995). The motivation to disconfirm the stereotype, or to avoid confirming it, represents pressure to succeed; however, high effort cannot always be invested. Instead, higher motivation to do well in stereotype-threatening situations can produce distracting and negative thoughts (Schmader et al., 2008).

According to Schmader et al.’s (2008) integrated process model of stereotype threat, when a negative stereotype becomes relevant to one’s performance, it triggers increased physiological arousal, which impairs working memory operations. Furthermore, stereotyped individuals are busy detecting self-relevant information and signs of failure, which is the second process that puts a strain on working memory. The last process is the suppression of negative thoughts and feelings resulting from the first two processes, which further consumes the working memory capacity necessary for successful performance. Taken together, all three processes described above lead to reduced working memory capacity in tasks requiring cognitive resources, which can lead to uncharacteristically poor performance on a test. In fact, Schmader and Johns (2003) found that priming negative stereotypes reduced women’s memory capacity. Furthermore, they determined that a reduction in working memory capacity mediated the effect of stereotype threat on women’s math performance. These results are in line with the idea that individuals experiencing stereotype threats have negative thoughts, which reduce their working memory capacity. Numerous studies conducted subsequently demonstrated negative cognition in stereotype threat situations, such as negative expectancies and thoughts (Cadinu et al., 2005) and task-related worry (Beilock et al., 2007; Gerstenberg et al., 2012). To concentrate on a test, these worry thoughts need to be ignored, which consumes cognitive resources.

Applied to the field of women in math, this means that if the stereotype that women are not as good as men in math becomes salient in a test situation, this stereotype activation draws women’s attention to task-irrelevant worry caused by the fear of conforming to the negative stereotype. Increased worry then reduces cognitive resources, thus degrading performance. In fact, in previous studies, women who were told that gender differences in math exist (i.e., stereotype threat) not only performed worse, but also reported having more negative thoughts about math compared with a control group (Cadinu et al., 2005; Beilock et al., 2007; Gerstenberg et al., 2012). In particular, Cadinu et al. (2005) asked a group of female university students to complete a difficult math test under stereotype threat or in a no-threat (control) condition. During the task, the women were asked to list any thoughts that came to their mind immediately before solving each of the seven difficult math problems. The authors predicted, *inter alia*, that individuals under stereotype threat would report more worry thoughts and show a decrease in performance compared with those under the control condition, and that worry would mediate the negative effects of stereotype threat on performance. As predicted, Cadinu et al.’s study found that women under stereotype threat reported more worry thoughts related to the test and showed a sharp decrease in performance compared with those in the no-threat condition. More importantly, performance degradation was mediated by an increase in worry. Therefore, they concluded that negative performance-related thoughts can consume working memory capacities to impede performance.

Similarly, Beilock et al. (2007) found that stereotype threat resulted in a greater proportion of task-related worry. Furthermore, this relation was attributed to the consumption of working memory resources. In another study conducted by Gerstenberg et al. (2012), female university students were asked about their current thoughts before the math test, but after the stereotype manipulation. They showed the highest level of worry thoughts (e.g., “I ask myself whether my performance will be good enough.”) when the stereotype threat was activated. Moreover, as has been shown by other researchers (e.g., Cadinu et al., 2005; Beilock et al., 2007), performance-inhibiting worry mediated the stereotype threat effect.

In summary, as Schmader et al.’s (2008) integrated process model suggests, if attention shifts away from the task, it is because people are having (negative) thoughts about their performance. Thus, in stereotype-threatening situations, distracting worry thoughts reduce the cognitive resources required to successfully elaborate task-relevant information.

### Self-Control Capacity in Test Situations

Self-control is defined as the mental capacity that enables people to override, inhibit, or modify their impulses, emotions, thoughts, and behaviors and to bring them in line with standards and personally endorsed overarching goals (e.g., Baumeister et al., 2007). Studies in different fields of psychological research have provided evidence that dealing with initial self-control demands briefly undermines an individual’s cognitive capacities that are required for subsequent working memory operations, regulating thoughts and emotions, and focusing attention in a goal-directed manner (Baumeister and Vohs, 2016). There is an ongoing debate regarding which mechanism underlies the effect of self-control-dependent performance decrements (e.g., Kurzban et al., 2013; Bertrams, 2020); in this regard, some authors have associated the detrimental effects of initial self-control on subsequent operations with mental fatigue or exhaustion (e.g., Job et al., 2010; Bertrams, 2020). It is also debated whether such an exhaustion effect of self-control exists at all (e.g., Englert and Bertrams, 2021). However, there is reasonable theory and empirical evidence for the existence of a varying self-control capacity, and many researchers have agreed that self-control cannot always be maintained in cognitively demanding situations (Baumeister and Vohs, 2016; Garrison et al., 2019; Bertrams, 2020; Dang et al., 2020).

In achievement tests, the exertion of self-control is required for cognitive processing as well as for focusing on the items’ content over longer periods while inhibiting distractions (e.g., negative thoughts). More precisely, executive functions [i.e., updating of working memory, inhibiting impulses, and shifting between mental sets; (Miyake et al., 2000)] seem to be key ingredients for successful self-control in achievement situations (Hofmann et al., 2012). Students with low compared with high levels of self-control capacity perform worse in working memory (Schmeichel, 2007), logical reasoning, and mental arithmetic tasks (Schmeichel et al., 2003).

Englert and Bertrams (2017) showed that eighth graders’ knowledge retrieval was undermined when their self-control capacity was briefly depleted in a previous unrelated self-control demanding task. This result indicates the relation between low self-control capacity and low working memory capacity, two ingredients that are required for working focused on item content, especially in science and math tests. In line with this assumption, Lindner et al. (2019) found that students with low working memory capacities showed an early onset of rapid-guessing behavior (i.e., unrealistic fast responses to test items) over the course of a science test, indicating a reduction in students’ test-taking efforts. In another study (Lindner et al., 2017), students with lower self-control capacity showed stronger progressive performance declines over the course of a computer-based mathematical problem-solving test compared with individuals with higher levels of self-control capacity. It can be assumed that the performance decrements were due to an increasing number of distracting thoughts, which could not be regulated effectively during the testing procedure.

As mentioned above, self-control capacity is also required for regulating negative emotions and thoughts. Consistent with this notion, Lindner and Retelsdorf (2020) found that students who perceived themselves as having lower levels of actual self-control capacity subsequently showed lower scores in an English as a foreign language test and reported more cognitive interruptions due to distracting thoughts. More directly related to threat and related worry during test situations, Bertrams et al. (2013) showed that test-anxious students who are more susceptible to experiencing threat were distracted more frequently by anxiety-related worry thoughts and, therefore, performed worse in an arithmetic test. However, the relational pattern among test anxiety, worry, and performance was found only in individuals whose self-control capacity had initially been experimentally impaired, not in individuals whose self-control capacity was intact.

### Present Research

Based on previous research, we assumed that the fear of stereotype confirmation can occupy the capacity of the cognitive system, and stereotype-threatening situations might trigger distracting and negative thoughts (Schmader et al., 2008). However, stereotype threat during evaluative situations should be more strongly related to distracting worry thoughts when the self-control capacity is momentarily lower compared with being intact. This pattern resembles Bertrams et al.’s (2013) findings on test anxiety, self-control capacity, and worry, as test anxiety is considered to be associated with the experience of threat (Spielberger and Vagg, 1995). However, unlike the existing research, we examined the effect of another cause of threat (i.e., being stereotyped) than trait test anxiety on worry with regard to self-control capacity.

More precisely, we examined the moderating influence of self-control capacity on gender stereotype threat in predicting the development of worry thoughts prior to and during an evaluative math test. For this reason, we invited female and male students, all of whom studied math at a German university, to the lab. The self-control capacity was impaired in the experimental condition and left intact in the control condition. In addition, we induced stereotype threat for all female but not male participants through standardized test instruction. Then, the participants reported their thoughts three times (after the stereotypical test instruction and after each half of a brief alleged test of mental arithmetic abilities). We assumed that being female is associated with higher worry in the present performance situation (Cadinu et al., 2005). However, based on previous research (Bertrams et al., 2013), we predicted that the relation between gender and worry would be more pronounced when self-control capacity is low compared with high.

## Materials and Methods

### Participants

Overall, 104 undergraduates enrolled in studies related to math (business mathematics or teaching training for math in schools) at a German university participated. None of them correctly guessed the true purpose of this study, but two indicated that they did not speak German fluently. As all materials in this study were presented in German, we decided prior to the analyses not to include their data. Thus, the final sample comprised 102 students (58% female; *M*_age_ = 21.70, SD_age_ = 2.29). The participants were randomly assigned to either the low (*n* = 50; *n* = 29 female, 21 male) or the high (*n* = 52; *n* = 30 female, 22 male) self-control capacity groups. The sample size decision was based on Simmons et al.’s (2011) recommendation to collect at least 20 participants per group, a recommendation that was a common guideline at the time the present data were collected (i.e., 2014).

Negative stereotypes about math abilities might not affect individuals who are not skilled or to whom math is essentially unimportant (Steele, 1997). However, the participants’ answers on their math skills (*M* = 6.13, SD = 1.33) and personal importance to be good at math (*M* = 6.51, SD = 1.88) indicated sufficiently high math-related skills and relevance, as they were above the midpoint of nine-point scales (Beilock et al., 2007). Moreover, the mean final grade from the secondary school (German Abitur; *M* = 1.82, SD = 0.54) indicated a high cognitive ability in this sample, given that it strongly diverged from the average Abitur grade in Germany in each of the 15 years from 2006 to 2020 (means interval = [2.37, 2.52], *p*s < 0.001, *d*s > 1.02, one-sample *t*-tests; note that lower numbers indicate higher performance in the German grading system; the German average Abitur grades were retrieved from Sekretariat der Ständigen Konferenz der Kultusminister der Länder in der Bundesrepublik Deutschland, 2022, January 7).

### Procedure

Participation lasted 25–30 min and occurred in a university laboratory room. After giving informed consent, the participants completed a “questionnaire about mathematics,” which included the measure of math-related trait test anxiety, one item on self-rated math ability, and another item on the personal relevance of math. Next, the manipulation of self-control capacity was performed. After that, the participants answered the manipulation check, a measure of self-competence, and a mood scale. This was followed by instructions for the math test and an explanation of the thought protocols. The explanation of the thought protocols also included the induction of stereotype threat for the female participants. Subsequently, the participants were asked to fill in the first thought protocol. After that, they worked on a brief math test that was interrupted by a second thought protocol. After the second part of the math test, the participants completed a third thought protocol. Then, the participants answered a questionnaire on their demographic data. The questionnaire also included items on their motivation to perform well during the math test as well as questions on the school-leaving grade, German-language ability, and hypothesis suspicion. Finally, the participants were thanked, debriefed, and either received course credit or €4 in exchange for their participation.

### Materials

#### Manipulation of Self-Control Capacity

We applied a manipulation task from previous research (e.g., Bertrams et al., 2010; Dummel and Rummel, 2016; Wiesner and Lindner, 2017). All participants were asked to transcribe a historical text about a German city. The text was free from threat-related content (e.g., battles, war, and fear). While the high self-control capacity group transcribed the text as it was (i.e., without further instructions), the low self-control group was instructed to always omit the frequent letters “e” and “n.” Thus, only the participants in the latter group had to volitionally override their elaborated writing habits and reduced their momentary self-control capacity by this self-control exertion. An English translation of this task is provided in the supplemental material of Bertrams et al. (2015). The experimenter stopped the participants after 6 min and asked them to put the sheet in a prepared concealing desk tray to remain blind to the experimental condition.

#### Induction of Stereotype Threat

All participants received a sheet explaining how to work on the thought protocols. The stereotype threat for the female participants was integrated into this instruction. It was claimed that the present research was about the cognitive processes that might explain why males have ostensibly been found to consistently perform better in math than females in the educational field, as well as in standardized lab tests. Thus, the instruction highlighted gender differences to the disadvantage of females. Such procedures have been extensively used to induce stereotype threat (e.g., Cadinu et al., 2005; Beilock et al., 2007).

#### Math Performance Situation

The participants received a single sheet presenting 20 arithmetic tasks in a row on the left. Each task comprised an initial subtraction, followed by a division. Examples are “(43–27): 8 =,” “(41–23): 4 =,” and “(43–27): 7 =.” Next to each task were 18 columns for indicating the solution by checking one box: “1,” “1 with remainder,” “2,” “2 with remainder,” … “9 with remainder.” We used this unusual arithmetic performance task, as this study did not focus on performance but on worry thoughts. Therefore, we made an effort to standardize the performance situation as much as possible (e.g., by making it unlikely that participants would use and focus on notes to facilitate mental calculation). The instruction for the performance task was to work as quickly as possible, as the working time was limited to 90 s, as well as to avoid wrong solutions. There were two performance blocks (i.e., two single sheets with 20 tasks each) located among the three thought protocols. For each block, the participants were stopped by the experimenter after 90 s.

#### Worry During the Math Test (Thought Protocols)

The participants were asked to write down on a lined sheet anything they could remember that went through their mind during the last half minute (first thought protocol)/while working on the block of math tasks they had just worked on (second and third thought protocols). Two independent judges rated the number of test-related worry thoughts mentioned in each thought protocol (Cadinu et al., 2005). The inter-rater reliabilities for the number of worry thoughts reported in the first, second, and third thought protocols were satisfying: *ICC*s = 0.78, 0.90, and 0.87, respectively, all *p*s < 0.001. The same was true for inter-rater reliabilities regarding the provided overall number of thoughts: *ICC*s = 0.96, 0.95, and 0.94, respectively, all *p*s < 0.001. Therefore, we averaged the two judges’ counts for each measurement time. Conforming with previous research (e.g., Beilock et al., 2007), we adjusted each individual’s number of worry thoughts to the individual overall number of thoughts separately for each measurement time. The resulting proportions were the three dependent variables of the extent of worry at the three measurement times.

We also asked the participants to write next to each thought the percentage of the overall time they spent on the respective content. This explorative measure, to the best of our knowledge, has not been applied in the past. As we received estimates that exceeded 100%, we had doubts about the usefulness of this measurement and decided not to analyze the respective responses further.

#### Self-Reports

We applied a brief version of the Test Anxiety Inventory-German (Wacker et al., 2008). With nine items, the susceptibility to experience of worry and emotionality during math test situations was assessed (e.g., “I am thinking about the consequences of possible failure,” and “My heart is pounding.”). The answers were given on a four-point Likert scale ranging from *almost never* (1) to *almost always* (4). McDonald’s omega was found to be 0.86.

The manipulation check comprised three items that have been previously used (Bertrams and Englert, 2014): “How effortful did you find the transcription task?,” “How difficult did you find it to execute the transcription task?,” and “How much did you suppress your usual writing habits during the transcription task?” McDonald’s omega across these three items was 0.58. In addition, the participants indicated on one item their self-perceived success regarding the transcription task (Bertrams and Englert, 2014): “How much did you succeed in performing the transcription task?” This measure was used to estimate whether the task could unintentionally influence perceptions of self-competence. These four items were completed on a seven-point Likert scale ranging from *not at all* (1) to *very much* (7).

The participants also completed the 10-item Positive and Negative Affect Schedule (Mackinnon et al., 1999). Five items were momentary positive affect (e.g., “inspired”), and the other five items were momentary negative affect (e.g., “afraid”) measured on a scale ranging from *not at all* (1) to *extremely* (5). In the present study, McDonald’s omega was 0.74 for positive affect and 0.64 for negative affect.

With two items obtained from Beilock et al. (2007), self-perceived math skills (“I am good at math.”) and the importance of math (“It is important to me that I am good at math.”) were measured at the beginning of the experimental procedure. Answers were given on a scale ranging from *does not apply at all* (1) to *does completely apply* (9). Moreover, to estimate overall academic skills, we asked for the average final grade from secondary school (German Abitur), with 1.0 as the best possible grade in Germany. Math achievement is an integral part of the German final secondary school grade.

For each of the two performance blocks, the participants indicated their motivation behind the respective performance block on two items (“I made an effort to solve as many math problems as possible,” and “I have made an effort to avoid incorrect answers.”). The participants responded to the items on a seven-point Likert scale ranging from *does not apply at all* (1) to *does completely apply* (7).

## Results

### Preliminary Analyses

With a series of two-way ANOVAs, we performed several comparisons among the four groups, resulting from crossing gender (female vs. male) with the self-control capacity condition (low vs. high). In the present specific sample of math students, math-related trait test anxiety did not vary in dependence on gender, self-control capacity condition, or their interaction (*p*s > 0.12). Therefore, we did not incorporate trait test anxiety into the model structures we empirically tested. Furthermore, there were no differences with respect to gender, self-control capacity condition, or their interaction in the self-reported motivation during the performance blocks (*p*s > 0.48).

As revealed by another two-way ANOVA with the factors gender and self-control capacity condition, the manipulation check indicated that the participants in the low compared with the high self-control capacity condition exerted more self-control during the transcription task (*M* = 4.03, SD = 1.11 vs. *M* = 2.85, SD = 0.99, *F*[1, 98] = 29.56, *p* < 0.001, $\eta_{\mathrm{part}}^{2}$ = 0.23). There were no group differences in the manipulation check as a function of gender or the interaction between gender and the self-control capacity condition (*p*s > 0.28). As expected, there was no difference in positive or negative mood immediately after the self-control capacity manipulation in dependence on gender, self-control capacity condition, or their interaction (*p*s > 0.18; two-way ANOVA). These results suggest that the self-control capacity manipulation was successful, while it did not unintentionally affect mood.

Diverging from previous research (e.g., Bertrams et al., 2013), a two-way ANOVA yielded an unexpected interaction between gender and the self-control capacity condition with respect to the item responses on self-perceived competence during the transcription task (*F*[1, 98] = 8.00, *p* = 0.006, $\eta_{\mathrm{part}}^{2}$ = 0.08). There was also a main effect of the self-control capacity group (*F*[1, 98] = 6.25, *p* = 0.01, $\eta_{\mathrm{part}}^{2}$ = 0.06), but no main effect of gender (*p* = 0.20). To interpret the significant interaction, we conducted independent samples *t*-tests. For female university students, self-perceived competence was lower in the low compared with the high self-control capacity condition (*M* = 4.28, SD = 1.07 vs. *M* = 5.47, SD = 1.04, *t*[57] = −4.34, *p* < 0.001, *d* = −1.13). This was not true for male university students (*M* = 4.62, SD = 1.02 vs. *M* = 4.55, SD = 1.34, *t*[41] = 0.20, *p* = 0.84, *d* = 0.06). Moreover, female compared with male university students reported higher perceived competence in the high self-control capacity condition (*M* = 5.47, SD = 1.04 vs. *M* = 4.55, SD = 1.34, *t*[50] = 2.80, *p* = 0.007, *d* = 0.79), but not in the low self-control capacity condition (*M* = 4.28, SD = 1.07 vs. *M* = 4.62, SD = 1.02, *t*[48] = −1.14, *p* = 0.26, *d* = −0.33). However, perceived competence was neither for female nor for male university students within any of the self-control capacity groups correlated with worry at any time of measurement (*p*s > 0.15). Moreover, including perceived competence as covariate did not change the findings obtained for the more parsimonious model without this covariate (see “Main Analyses”). This suggests that any relation between gender and worry was not attributable to the possibility that the transcription task caused lasting group differences in perceived competence.

### Main Analyses

**Figure 1:**
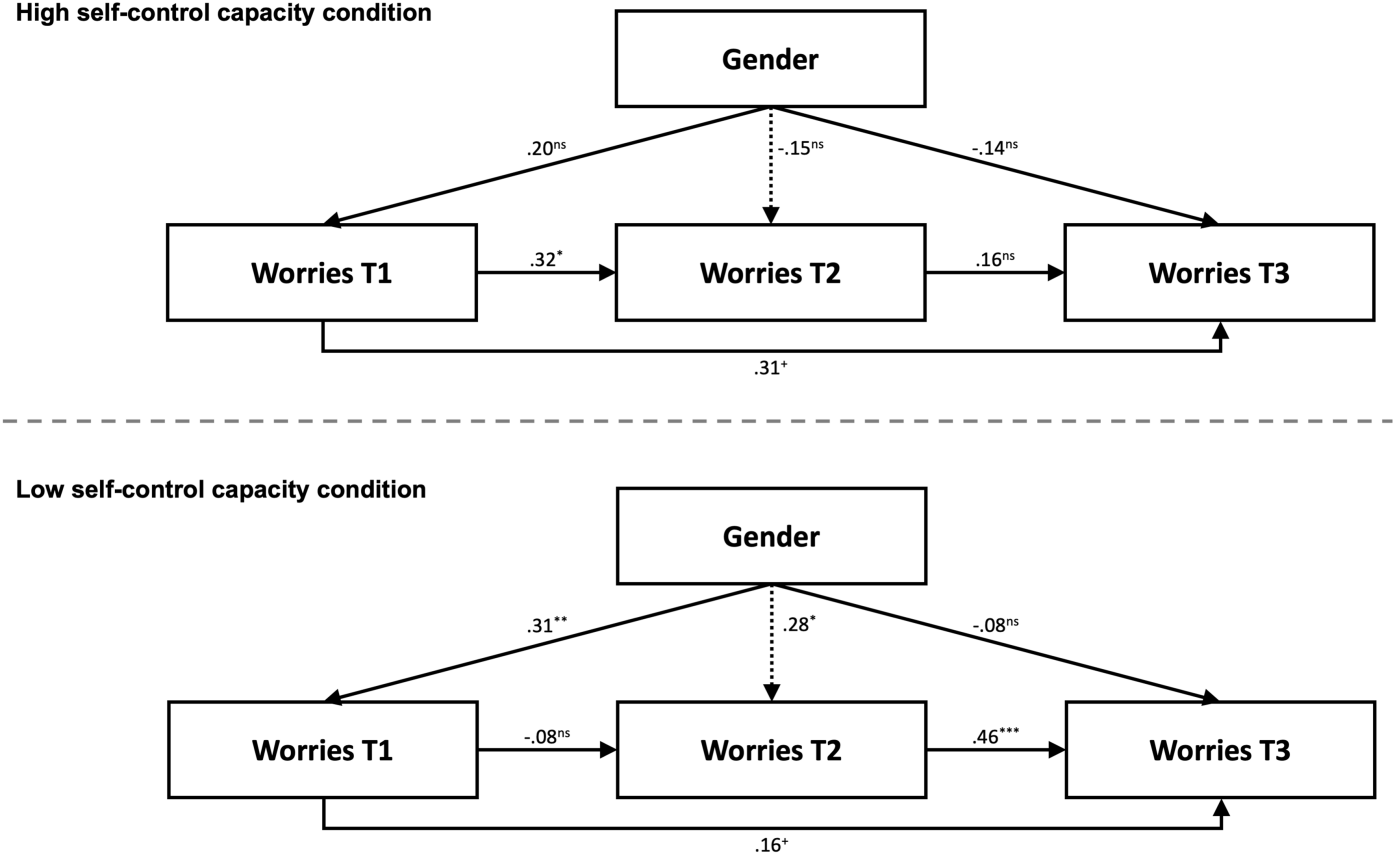
Multiple-group autoregressive path model including gender as a covariate. Top: High self-control capacity condition. Bottom: Low self-control capacity condition. Coding of gender: 0 = male, 1 = female. T1/T2/T3 = first/second/third measurement times. Presented are standardized path coefficients. Dotted lines differ significantly between the two conditions. *N* = 102. ^***^p < 0.001, ^**^p < 0.01, ^*^p < 0.05, ^+^*p* < 0.10, ns = nonsignificant.

To test our hypotheses regarding the prediction of worry by gender in different experimental conditions, we relied on multiple-group autoregressive path models. Therefore, we estimated the model depicted in Figure 1. This approach allowed us to directly test the moderation of the path coefficients from gender to the three worry variables at each time point by the experimental condition (low vs. high self-control capacity). Therefore, we applied the Wald test for each standardized path coefficient to determine whether it differed significantly between the two experimental conditions, indicating a moderation of the path by the participants’ state of self-control capacity. As our model comprised manifest variables and all possible relations between the variables were allowed, our model was saturated with *df* = 0, and no model fit statistics could be calculated. The model was estimated using M*plus* 8.4 (Muthén and Muthén, 2017) and applying a robust maximum likelihood estimator.

The means and standard deviations for the worry measures are presented in Table 1. As shown in Figure 1, in the high self-control capacity condition, there were no significant relations between gender and worry at any measurement time. In contrast, in the low self-control capacity condition, gender significantly predicted worry at the first and second measurement times (i.e., female university students reported more worry than male university students), but not at the third time. The path from gender to worry at the second measurement time (i.e., immediately after the first 90-s experience with the performance situation) was significantly different between the low and high self-control capacity conditions (Table 2).

**Table 1:** Means and standard deviations (in parentheses) of reported worry at the three times of measurement, separated by self-control capacity condition and gender.

	Self-control capacity high		Self-control capacitylow	
	Variable	Females	Males	Females	Males
Worry at T1	0.38 (0.31)	0.25 (0.33)	0.43 (0.30)	0.25 (0.23)
Worry at T2	0.53 (0.35)	0.59 (0.37)	0.60 (0.31)	0.46 (0.24)
Worry at T3	0.38 (0.34)	0.45 (0.34)	0.39 (0.25)	0.34 (0.31)

*N = 102. Worry = Proportion of the number of worry thoughts in the total number of thoughts. T1/T2/T3 = first/second/third measurement times*.

**Table 2:** Significance of differences in standardized path coefficients between the high and low self-control capacity conditions.

Path	Wald *χ*^2^	*df*	*p*
Gender ➔ Worry T1	0.38	1	0.54
Gender ➔ Worry T2	4.63	1	0.03
Gender ➔ Worry T3	0.12	1	0.73
Worry T1 ➔ Worry T2	3.44	1	0.06
Worry T1 ➔ Worry T3	0.56	1	0.46
Worry T2 ➔ Worry T3	2.15	1	0.14

*N = 102. T1/T2/T3 = first/second/third measurement times*.

### Auxiliary Analyses

In the high self-control capacity condition, the worry measures at the different measurement times were significantly related to each other, except for the worry at the second and third measurement times (Figure 1). This was different in the low self-control capacity condition, where worry at the first measurement time was not significantly related to worry measures at the second and third times. However, the moderation of these relations among the worry measures by the self-control capacity condition was not significant (Table 2).

**Figure 2:**
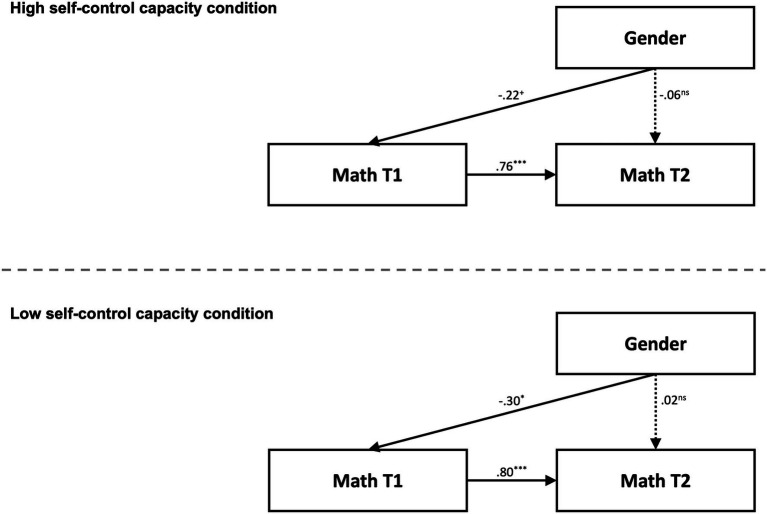
Multiple-group autoregressive path model including gender as a covariate. Top: High self-control capacity condition. Bottom: Low self-control capacity condition. Coding of gender: 0 = male, 1 = female. T1/T2 = first/second measurement times. Presented are standardized path coefficients. Dotted lines differ significantly between the two conditions. *N* = 102. ^***^p < 0.001, ^**^p < 0.01, ^*^p < 0.05, ^+^*p* < 0.10, ns = nonsignificant.

We also applied multiple-group autoregressive path models to examine whether performance was predicted by gender in different experimental conditions (see the model depicted in Figure 2). In contrast to the high self-control capacity condition, there was a significant path from gender to performance at the first measurement time in the low self-control capacity condition (Figure 2), suggesting that the performance of female compared with male university students was lower only when self-control capacity was low. However, this path as well as the other paths in the model were not significantly different between the two self-control capacity conditions (Table 3). In neither of the two self-control capacity conditions was gender predictive of performance at the second measurement time.

**Table 3:** Significance of differences in standardized path coefficients between the high and low self-control capacity conditions.

Path	Wald *χ*^2^	*df*	*p*
Math T1 ➔ Math T2	0.11	1	0.75
Gender ➔ Math T1	0.15	1	0.70
Gender ➔ Math T2	0.46	1	0.50

*N = 102. T1/T2 = first/second measurement times*.

## Discussion

### Present Findings

The present experiment aimed to show that situational differences in the capacity to exert self-control can determine how strongly stereotype threat is associated with worry in female university students during math test situations. We found some supporting evidence for this prediction; however, the pattern we obtained requires closer examination. In line with our assumption and previous studies on test anxiety (Bertrams et al., 2013), the only significant paths from gender to the extent of worry emerged in the experimental condition in which the self-control capacity was relatively low. In this case, female university students reported more worry than their male counterparts. This is unsurprising, as the female but not the male participants in our study received threatening instruction with respect to the math performance situation. In contrast, we did not find any significant gender–worry relations in the high self-control capacity condition. This finding can be interpreted such that stereotype threat has adverse effects on cognition, as has been theorized and empirically demonstrated in the past (Cadinu et al., 2005; Schmader et al., 2008). However, such stereotype threat effects are controllable and repressible. When their self-control capacity is intact, individuals can deliberately focus their stream of thought on ongoing tasks, also directing them away from threat-related worry. This effect was mostly pronounced in the middle of the performance situation (i.e., at the second measurement time). For the worry that was reported between the two performance blocks and referred to the first experience with the math tasks, the relation between gender and worry was significantly different for the two self-control capacity conditions.

The experimental conditions did not significantly differ in the relation between gender and worry at the first measurement time, even though the significance and non-significance of this relation in the low and high self-control capacity conditions, respectively, confirmed our expectations. In the high self-control capacity condition, the female university students could initially have experienced some threat that did not build up to a full stereotype threat effect but nevertheless increased the gender–worry relation. Note that at the first measurement time, the students had already been confronted with the stereotype instruction but had not yet seen the math tasks; therefore, uncertainty about what to expect is likely to have contributed to a threat experience. As a result, the difference between the two experimental conditions in the model path at the first measurement time might not have been large enough to reach statistical significance. The influence of uncertainty then vanished after the math tasks had been received, leading to a significant difference in the stereotype threat effect at the second measurement time.

Even in the low self-control capacity condition, the threatened female participants did not experience more worry than the non-threatened male participants at the third measurement time. There are at least two possible explanations for this finding. First, the third thought protocol was placed after the second performance block (i.e., when the performance situation had already ended). Although the instruction for the thought protocol was to report the thoughts during the second math block, it seems reasonable to assume that some worried female participants were relieved about the end of the performance situation. This relief might have biased the third thought protocol. Second, the stereotype threat effect could have declined during the math performance situation due to factors such as habituation. Similarly, in Cadinu et al.’s (2005) study, the relation between stereotype threat and worry during the second half of a math performance situation was relatively low compared with the first half. At the moment, we cannot determine whether the lack of a stereotype threat effect at the third measurement time was caused by a methodological problem or represents a typically occurring pattern.

We obtained an interesting pattern of relations for the stabilities between the worry measures at different measurement times. In the high self-control capacity condition, the worry measures at the first but not the second measurement time predicted worry at the third measurement time. One explanation is that worry at the second measurement time did not incrementally add even more worry beyond the amount of worry at the first measurement time. However, why was worry at the first measurement time unrelated to the worry measures at the other two times in the low self-control capacity condition? Unfortunately, we cannot answer this question satisfactorily, but suspect that a qualitative shift in worry occurred as a result of contact with the math tasks of the first block. Over and above the stereotype threat, some individuals could have been perturbed just by the announcement of a performance situation. Given their momentary lack of self-control capacity to regulate their negative experiences, this might have impacted their reports of worry regarding the period during and immediately after the announcement (i.e., the thought protocol at the first measurement time). After the first confrontation with the performance situation was made (i.e., at the second measurement time), this influence of diffuse expectations might have ceased. Instead, at the second measurement time, the participants’ perception of how they were going to actually deal with the performance situation determined the worry. Thus, some individuals may have experienced the actual performance situation as less alarming than expected, and vice versa; others may have seen their expectations as confirmed, overall resulting in the lack of a relation between the worry measures at the first and second measurement times.

### Implications

The present findings extend knowledge about the moderating situational circumstances under which stereotype threat can be harmful. Exerting self-control during an unrelated demand can apparently undermine personal resistibility against self-threatening information in subsequent performance contexts. Therefore, it is advisable not to place self-control requirements in advance of important performance situations, particularly for individuals who are at risk of being stereotyped, as we recreated in the present study for females in the context of math. Possibly, similar effects are at work for males in the context of language-related tasks (Bedyńska et al., 2021; but see Chaffee et al., 2020). Viewed from a positive perspective, the present findings also indicate that stereotyping information may not, in any case, be irritating for the individuals concerned. Momentary self-control capacity might thus be a moderator variable, which can explain why there are numerous studies that found stereotype threat effects, while others did not replicate this finding (e.g., Chaffee et al., 2020; McGuire et al., 2021). In general, unconsidered moderator variables have been argued to be one crucial reason for inconsistent findings (e.g., Bertrams et al., 2013; Stroebe and Strack, 2014).

Furthermore, this study complements the research on self-control capacity and test anxiety (Bertrams et al., 2013). It was previously found that the relation between anxiety (instead of stereotype threat) and worry during a performance situation was moderated by momentary self-control capacity. When self-control capacity was relatively intact, trait test anxiety and worry during the performance situation were unrelated; however, when the self-control capacity was reduced by an unrelated previous self-control demand, higher trait test anxiety was associated with a more pronounced experience of distracting worry in the subsequent performance situation. Thus, across different studies, we found a pattern that was largely consistent across various threats (i.e., threat caused by trait test anxiety as a personality variable and threat caused by stereotype activation for a specific group). In this regard, in the present study, trait test anxiety did not differ among gender, self-control capacity condition, or their combination. In summary, there is accumulating evidence that self-control is important in potentially threatening performance contexts.

### Limitations and Future Research

Differences in perceived competence that we unexpectedly found in dependence of gender and self-control condition may constitute an alternative explanation for our results. However, when incorporated as covariate, perceived competence did not change the findings. In addition, perceived competence did not predict worry at any time of measurement for any combination of gender and self-control capacity condition. Therefore, we assume that our results were not caused by differences in perceived competence. Still, further in-depth research may shed more light on the role of perceived competence. In this respect, it may be relevant that previous research has usually revealed no effects of the applied self-control capacity manipulation on perceived competence (e.g., Bertrams et al., 2013), but there is also initial evidence that self-control capacity manipulations affected self-efficacy (Graham et al., 2017), a variable that has some conceptual overlap with perceived competence.

In this study, we focused on a direct effect of the presence of a gender stereotype: worry (Schmader et al., 2008). For this reason, we embedded our dependent variable measure into a performance situation with math tasks. The presentation of these tasks was intended to frame the worry measures. Therefore, the math tasks were highly standardized in two blocks with an unusual response format and applied within a short time limit. In other words, our primary interest was not to find a reliable and valid performance measure. Actually, we think that the applied performance task is not appropriate or useful to draw conclusions about effects on real performance, for instance, because the time limit was much too short to represent a test with sufficient construct validity (see Lu and Sireci, 2007). While the chosen procedure may have been useful for measuring and analyzing the central variable of worry, it also implies a limitation of our study, namely, that we could not determine the impact of stereotype threat on performance. Indeed, no such empirical evidence was found in the present study. In future studies, the effects of a combination of self-control capacity and stereotype threat on performance should be addressed directly. In this regard, we think that measuring performance while thought protocols are embedded would not be ideal, as the interruptions may have uncontrolled effects on performance measurement. Instead, we recommend using a multistep experimental approach to detect a causal chain (Spencer et al., 2005).

Given the means of the worry measures, it cannot be ruled out that self-control capacity moderated a stereotype lift effect (Walton and Cohen, 2003), rather than a stereotype threat effect. Possibly, the stereotype threat instruction we used may have increased confidence and reduced worry in the male participants. At the moment, however, we cannot provide a theoretical basis beyond pure speculation as to why low self-control capacity would facilitate the stereotype lift effect. Basically, the assumption that low compared to high self-control capacity would be associated with lower worry during test situations contradicts previous reasoning (e.g., Bertrams et al., 2013). The potential interplay between self-control capacity and stereotype lift could still be a promising subject for future examination.

The present research can also be challenged by the ongoing debate on whether individuals’ self-control capacity is actually reduced after initial self-control exertion. Some authors deny the view that initial self-control detrimentally affects subsequent self-control and cognition (e.g., Carter et al., 2015), while others defend it (e.g., Baumeister et al., 2020; see also Englert and Bertrams, 2021). We believe that discussing and investigating the underlying mechanisms and potential moderator variables regarding situational fluctuations in self-control capacity is an appropriate way forward (e.g., Bertrams, 2020, 2021). In the present study, we found an effect of our manipulation of self-control capacity on worry; however, there might be different ways to interpret it. For instance, the manipulation might have undermined self-control by exhausting a self-regulatory resource (Baumeister et al., 2007) or by activating the cognitive concepts of fatigue and energy saving (Bertrams, 2020). Future research can determine in more detail when and how self-control exertion can harm subsequent self-control. These insights can advance the understanding of our findings and how interventions based on them can be optimally designed.

To conclude, the results from the present study represent a step toward understanding the conditions of females’ underachievement in math under stereotype threat. By explicitly showing the moderating function of students’ momentary self-control capacity in the relation between stereotype threat and worry, our study contributes to a deeper understanding of the mechanisms of stereotype threat.

## Data Availability Statement

The raw data supporting the conclusions of this article will be made available by the authors, without undue reservation.

## Ethics Statement

Ethical review and approval was not required for the study on human participants in accordance with the local legislation and institutional requirements. The patients/participants provided their written informed consent to participate in this study.

## Author Contributions

AB developed the study concept and design and collected the data. AB and JR analyzed and interpreted the data. AB, CL, FM, and JR wrote the manuscript. All authors approve the final version to be published, contributed meaningfully to the paper, and agree to be accountable for all aspects of the work in ensuring that questions related to the accuracy or integrity of any part of the work are appropriately investigated and resolved.

## Funding

Open access funding provided by University of Bern.

## Conflict of Interest

The authors declare that the research was conducted in the absence of any commercial or financial relationships that could be construed as a potential conflict of interest.

## Publisher’s Note

All claims expressed in this article are solely those of the authors and do not necessarily represent those of their affiliated organizations, or those of the publisher, the editors and the reviewers. Any product that may be evaluated in this article, or claim that may be made by its manufacturer, is not guaranteed or endorsed by the publisher.
